# Supplementary material for: Surface Plasmon Resonance Assay for Label-Free and Selective Detection of HIV-1 p24 Protein
Source: Biosensors (Basel). 2021 Jun 3;11(6):180. doi: 10.3390/bios11060180 (PMC8229864; doi:10.3390/bios11060180)
Supplement: Supplementary file 1 [file biosensors-11-00180-s001.zip › biosensors-1230421-supplementary.pdf]

Supporting information

Surface Plasmon Resonance assay for label-free and selective detection of HIV-1 p24 Protein

Lucia Sarcina [1], Fabrizio Torricelli[2], Paolo Bollella,[1] Zahara Gounani,[3] Ronald Österbacka,[3] Eleonora Macchia [3,*] and Luisa Torsi[1,3,4]

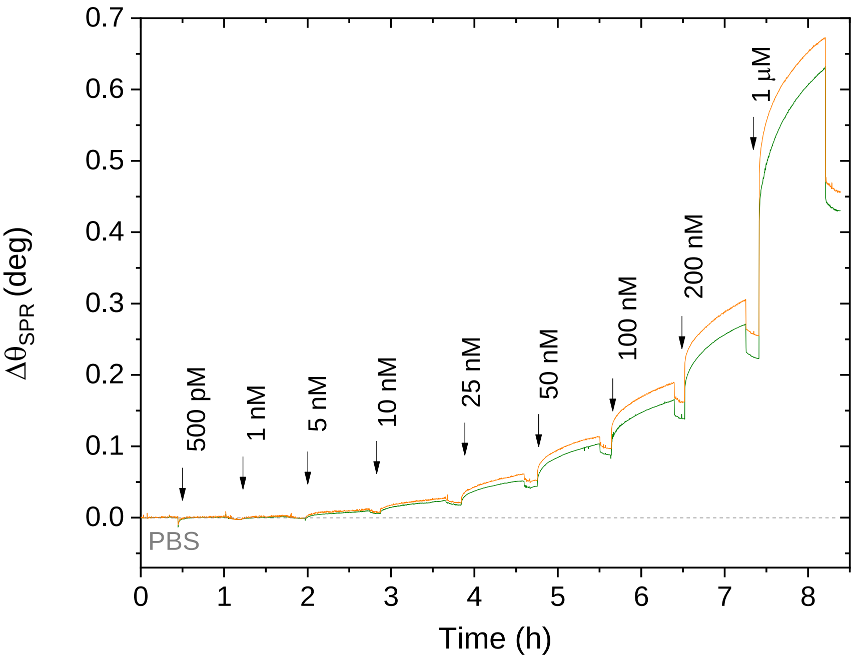

Figure S1. SPR sensogram recorded for the real-time exposure of the HIV-1 anti-p24 modified SAM to the target protein p24. The protein concentration ranged from 500 pM to 1uM. The baseline level was established in PBS.

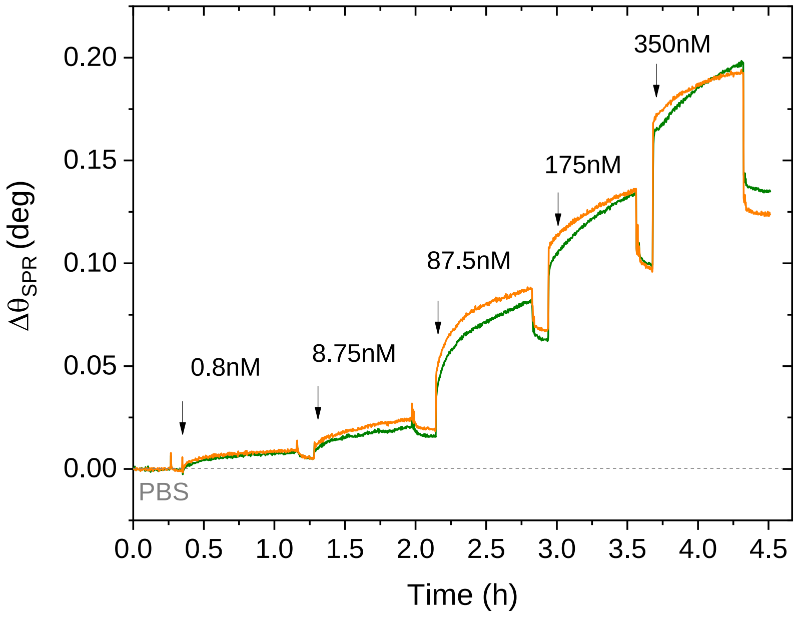

Figure S2. SPR sensogram of the assay of HIV-1 p24 protein at increasing concentrations (0.8 nM – 350 nM), performed on the physisorbed anti-p24 antibodies on gold.
